# Supplementary material for: The RNA-binding protein NOVA-1 regulates circRNA expression, alternative splicing, and aging in Caenorhabditis elegans
Source: G3 (Bethesda). 2026 Jan 22;16(4):jkag016. doi: 10.1093/g3journal/jkag016 (PMC13042279; doi:10.1093/g3journal/jkag016)
Supplement: jkag016_Supplementary_Data [file jkag016_supplementary_data.zip › Supplemental_Material_Legends_G3-2025-406454.docx]

**The RNA-binding protein NOVA-1 regulates circRNA expression, alternative splicing, and aging in *C. elegans***

Emmanuel Adeyemi ^1,#^, Hussam Z. Alshareef ^1,2,#^, Jaffar M. Bhat ^1,3^, Pedro Miura ^4,5^, and Alexander M. van der Linden ^1,*^

^1^ Department of Biology, University of Nevada, Reno, NV 89557, USA

^2^ Present address: Stiles-Nicholson Brain Institute, Florida Atlantic University, Jupiter, FL 33458, USA

^3^ Present address: Department of Botany, Government Degree College for Women Sopore, Jammu and Kashmir, 193201, India

^4^ Department of Department of Genetics and Genome Sciences, University of Connecticut School of Medicine, Farmington, CT 06030, USA

^5^ Institute for System Genomics, University of Connecticut, Storrs, CT 06269, USA

* Corresponding author: Department of Biology, University of Nevada, Reno, NV 89557, USA. Email: Alexander van der Linden: [avanderlinden@unr.edu](mailto:avanderlinden@unr.edu)

^#^ These authors contributed equally to the work.

**Supplementary Information**

**Supplementary Figure 1: (A)** Schematic of mutation in the *nova-1* gene used in this study. The allele was generated using CRISPR/Cas9 (see Material and Methods). **(B)** Sashimi plot illustrating differential splicing events between wild-type and *nova-1* mutants, involving exon 4 at the *chr-1* locus (chrIII:11688217-11691174). Filled peaks represent read coverage for each sample, and arcs indicate splice junctions with corresponding junction read counts. Top arcs display junction read counts for canonical splicing between exon 3 and 4 while bottom arcs display junction read counts for A3’SS selection. Light blue peaks and lines indicate exon splice sites and splicing events in wild-type samples; dark blue peaks indicate exon splice sites and splicing events in *nova-1* mutants. Sashimi plot generated using the shiba2sashimi (v0.1.7) package. **(C)** YCAY site summary in significant and non-significant A3’SS events between wild-type and *nova-1* mutants. The non-significant set includes >2,000 transcripts serving as negative controls, while 195 A3’SS events were classified as significantly regulated (FDR ≤ 0.05, |ΔPSI| ≥ 0.2). **(D)** Venn Diagram showing the overlap between 1,166 previously reported age-associated *C. elegans* circRNAs (Cortés-López et al. 2018) with the 103 *nova-1*-regulated circRNAs identified in this study.

**Supplementary Table 1: Oligonucleotide primers**

All primer sequences for linear or circular RNAs used in this study.

**Supplementary Table 2: RNA sequencing read statistics**

RNA-seq read statistics for 5 independent biological samples of wild-type and *nova-1(tm6146)* mutants with NCBI Sequence Read Archive (SRA) and NCBI Gene Expression Omnibus (GEO) IDs for each sample.

**Supplementary Table 3: Detected and differentially expressed circRNAs**

Tab 1 lists all differentially expressed (DE) circRNAs between wild-type and *nova-1(tm6146)* mutants. Tab 2 lists all detected circRNAs in the RNA-seq samples.

**Supplementary Table 4: Differential linear splicing events**

Shown are differential linear splicing events between wild-type and *nova-1(tm6146)* mutants. Tab 1 lists all differential alternative 3’ splicing events (A3’SS). Tab 2 lists all differential skipped exons (SE). Tab 3 lists all differential retained introns (RI). Tab 4 lists all differentia alternative 5’ splicing events (A5’SS). Tab 5 lists all differential exclusive exons (MXE). Tab 6 lists all non-significant A3’SS events. In all tabs, red color indicates enriched (>60) YCAY sites, orange indicates intermediate (20–59) YCAY sites, and light blue indicates low (<20) YCAY sites. The Reduced Percent Spliced In (PSI) is indicated for each splicing event in wild-type (WT) and *nova-1(tm6146)* mutants.

**Literature cited**

Cortés-López M, Gruner MR, Cooper DA, Gruner HN, Voda AI, van der Linden AM,

Miura P. 2018. Global accumulation of circRNAs during aging in *Caenorhabditis*

*elegans*. *BMC Genomics* **19**: 8.
