# Supplementary figures and images for: The RNA-binding protein NOVA-1 regulates circRNA expression, alternative splicing, and aging in Caenorhabditis elegans
Source: G3 (Bethesda). 2026 Jan 22;16(4):jkag016. doi: 10.1093/g3journal/jkag016 (PMC13042279; doi:10.1093/g3journal/jkag016)

Supplementary Figure 1

A

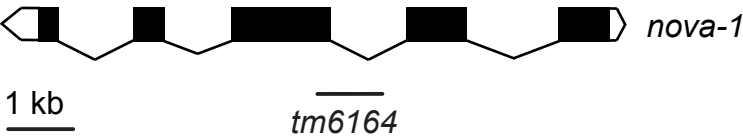

C

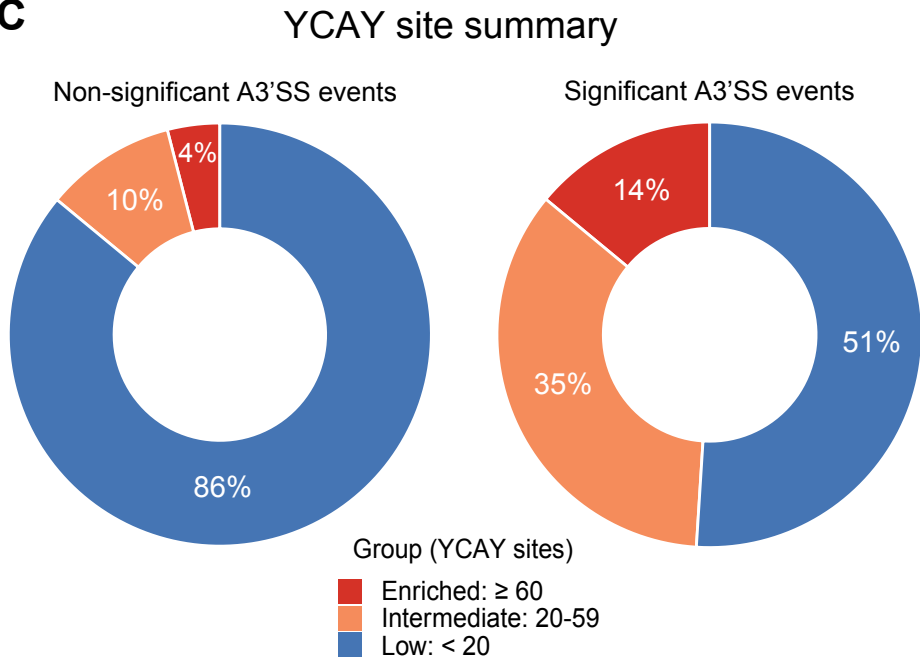

D

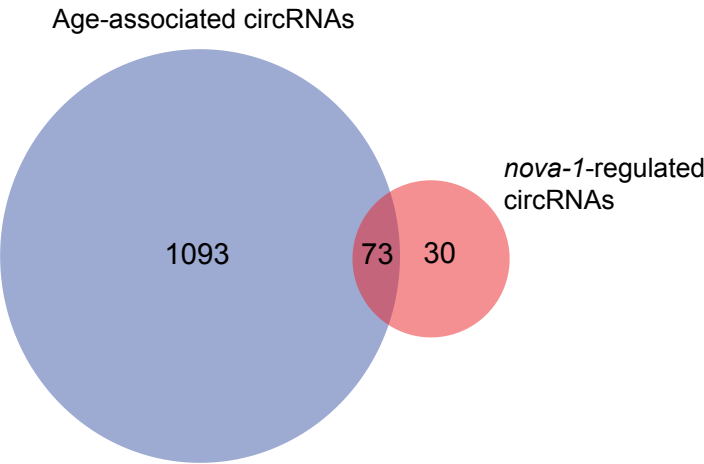

B

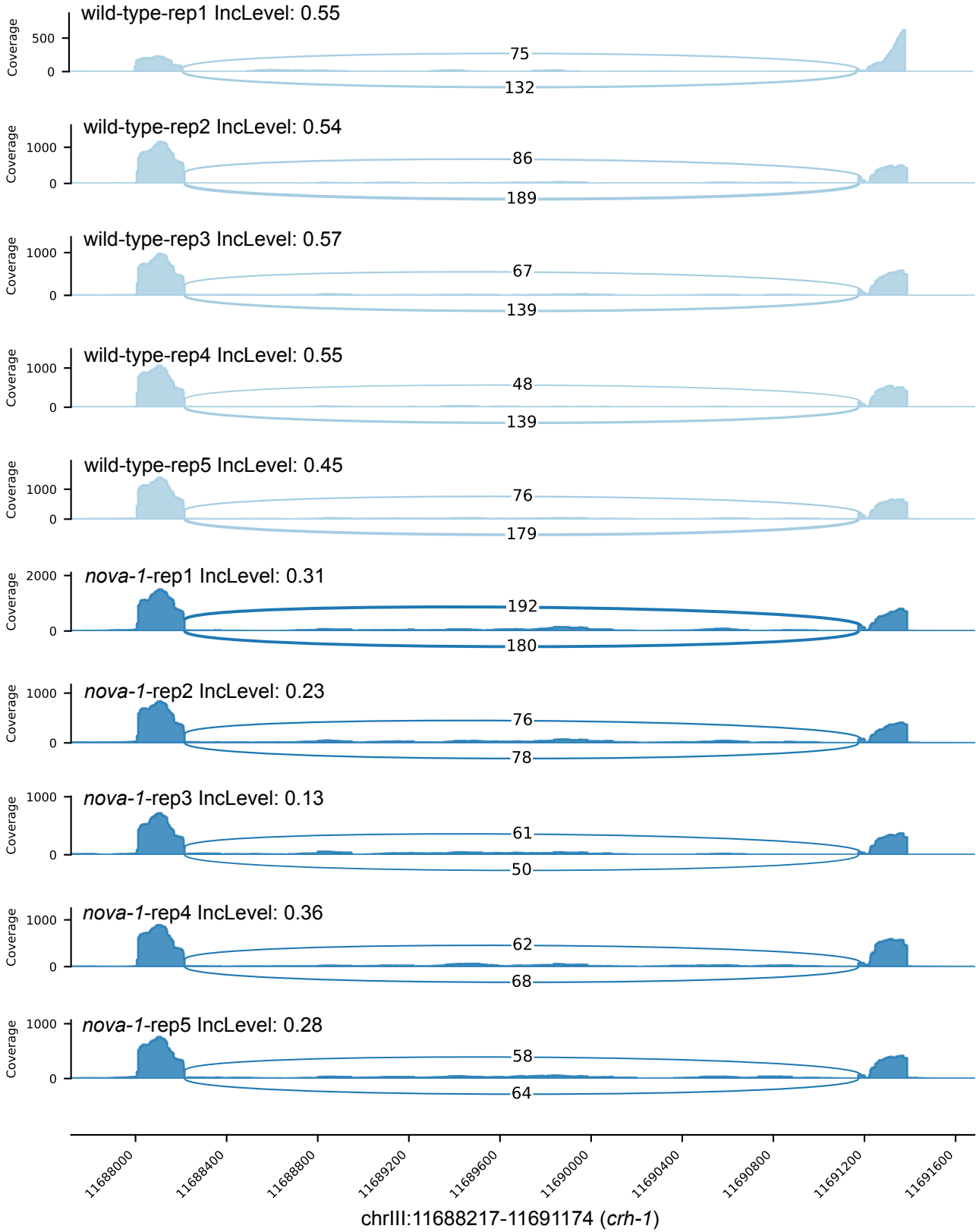

Supplement: jkag016_Supplementary_Data [file jkag016_supplementary_data.zip › Supplementary_Figure_1_G3-2025-406454.pdf]
